# Lower Limb Ischemia as Acute Onset of Primary Aortic Occlusion: CTA Imaging and Management

**DOI:** 10.3390/ijerph20053868

**Published:** 2023-02-22

**Authors:** Giulia Lassandro, Stefania Tamburrini, Carlo Liguori, Stefano Giusto Picchi, Filomena Pezzullo, Giovanni Ferrandino, Fabio Spinetti, Gennaro Vigliotti, Ines Marano, Mariano Scaglione

**Affiliations:** 1 Department of Radiology, Ospedale del Mare, ASL NA1 Centro, Via Enrico Russo, 80147 Naples, Italy; 2 Department of Vascular Surgery, Ospedale del Mare, ASL NA1 Centro, 80147 Naples, Italy; 3 Department of Medicine, Surgery and Pharmacy, University of Sassari, Piazza Università, 21, 07100 Sassari, Italy

**Keywords:** aorta, thrombosis, primary aortic occlusion, arterial occlusive disease, occlusion, vascular, ischemia, computed tomography angiography, embolism

## Abstract

Primary aortic occlusion (PAO) is defined as acute occlusion in the absence of aortic atherosclerosis or aneurysm. PAO is a rare disease with acute onset and can determine massive parenchymal ischemia and distal arterial embolization. The aim of our study was to focus on the assessment of clinical characteristic, CT signs, medical and surgical treatment, complication rates and the overall survival of PAO. Materials and Methods: We retrospectively analyzed the data of all patients with acute lower limb ischemia and a final surgical or discharge diagnosis of PAO who underwent aortic CT angiography in ER settings in our hospital from January 2019 to November 2022. Results: A total of 11 patients (8 males/3 females; male/female ratio, 2.66:1, age range 49 to 79 years-old, mean age 65.27 y/o) with acute onset of lower limb impotence or ischemia were diagnosed with PAO. The etiology was thrombosis in all patients. The aortic occlusion was always located in the abdominal aorta and extended bilaterally through the common iliac arteries. The upper limit of the thrombosis was detected in the aortic subrenal tract in 81.8% of the cases, and in the infrarenal tract in 18.2%. A total of 81.8% of the patients were referred to the ER for symptoms related to lower limb: bilateral acute pain, hypothermia and sudden onset of functional impotence. Two patients (18.2%) died before undergoing surgery for multi-organ failure determined by the severe acute ischemia. The other patients (81.8%) underwent surgical treatment that included aortoiliac embolectomy (54.5%), aortoiliac embolectomy + aorto-femoral bypass (18.2%) and aortoiliac embolectomy and right lower limb amputation (9.1%). The overall mortality was 36.4% while the estimated survival at 1 year was 63.6%. Conclusions: PAO is a rare entity with high morbidity and mortality rates if not recognized and treated promptly. Acute onset of lower limb impotence is the most common clinical presentation of PAO. Aortic CT angiography is the first-choice imaging technique for the early diagnosis of this disease and for the surgical treatment, planning and assessment of any complications. Combined with surgical treatment, anticoagulation is considered the first-line medical therapy at the time of diagnosis, during surgical treatment and after at discharge.

## 1. Introduction

Acute aortic occlusion (AAO) is a rare and potentially life-threatening disease [1,2,3] with mortality rates up to 75% [4]. AAO may have a primary or spontaneous etiology or be secondary to other causes [5]. The etiology of secondary aortic occlusion (SAO) is variable: embolism from the proximal aorta or the heart, progression of atherosclerotic lesions, acute occlusion of an abdominal aortic aneurysm, occlusion after previous endovascular aortic interventions, and graft thrombosis [2,4,5,6]. The thrombotic occlusion appears to be more frequent [4,7]. SAO has also been reported in patients with inflammatory bowel diseases, in cancer patients undergoing chemotherapy and recently in patients affected by COVID-19 [1,8,9,10,11].

Primary aortic occlusion (PAO) is an extremely rare event with an unclear incidence due to its infrequent occurrence [5]. The etiology of PAO is unknown because it occurs in absence of atherosclerosis or aneurismatic pathology, and data are largely based on cases [5,12,13,14]. Acute thrombosis in the arterial system is considered a relatively rare event and the etiology of thrombosis is multifactorial. The pathophysiologic elements, also known as the Virchow’s triad, leading to thrombosis include abnormalities in blood composition (hypercoagulability), abnormalities in vessel wall components (endothelial changes and/or endothelial injury) and blood flow anomalies (hemodynamic changes) [1,15]. The review of the literature suggests that the interaction between the host and environment factors can lead to acute thrombosis of the non-pathologic abdominal aorta, where hypercoagulability disorders, immunologic and metabolic factors and toxicological cofactors may play an important role [1,16]. PAO usually affects the distal abdominal aorta, subrenal or proximity to the aortoiliac bifurcation [2,3,17].

Clinical features depend on the level of occlusion; PAO is characterized by the acute onset of symptoms that are determined by the level of occlusion, by the size of the aortic branches occluded and by the presence of collaterals available. PAO in the ascending and descending aorta is even more rare than in the abdominal aorta, and patients can present symptoms related to cerebral vessel involvement with transient ischemic attacks and stroke, or symptoms related to coronary involvement with angina and myocardial infarction [18]. In patients whose abdominal aorta is involved, symptoms are often related to sudden motor or sensory deficits of varying degrees. Acute onset of lower limb impotence, hypothermia and pain is the most frequent clinical picture because abdominal PAO usually involves aortic bifurcation and extends through the iliac–femoral axis [1,18]. Sudden paraplegia has also been reported, which is determined by the acute occlusion of the ostium of radicularis magna or Adamkiewicz artery and supplementary arterial ansa of the conus [18,19]. Furthermore, symptoms may relate to parenchymal ischemia [18].

Computed tomography (CT) is selected worldwide as the initial test in emergency settings for the diagnosis of acute aortic syndromes [20,21,22]. CT angiography can reach an accurate diagnosis rapidly and allow for near-isotropic voxels and high-quality coronal and sagittal multiplanar reconstructions, as well as three-dimensional (3D)-rendered maximum intensity projection (MIP) and shaded surface display images, all of which can aid in accurate diagnosis and surgical planning [22].

The treatment of PAO is controversial; anticoagulation has been considered the first-choice treatment, but the persistence of thrombus load or recurrent embolism risk has been reported in a high rate (>25%) compared with the open surgical removal of the thrombus (9%). In addition, it has been reported that recurrent embolism significantly increases the risk of major amputation (9% for anticoagulation alone vs. 2.3% for the surgical group) and life-threatening visceral ischemia [23]. For these reasons, anticoagulation treatment is nowadays initiated upon AAO diagnosis and continued during and after surgery, while exclusive medical treatment is performed on selected patients [17]. Surgical reperfusion via transfemoral thrombectomy is considered the first-line surgical treatment; axillo-bifemoral bypass is used in cases of inadequate revascularization. If the occlusion occurs in the infrarenal abdominal aorta, aortic reconstruction with axillo-bifemoral bypass and branched graft to visceral arteries is performed [4,7].

The aim of our study was to focus on the clinical picture, diagnosis and treatment of abdominal PAO.

## 2. Materials and Methods

### 2.1. Patients

A retrospective analysis was performed on the medical and surgical records of patients who were diagnosed with primary aortic occlusive thrombosis who were referred to our hospital in emergency settings from January 2019 to November 2022.

Two radiologists (ST and GL) independently reviewed the CTA exams on a PACS workstation (CARESTREAM), blinded to the identity, clinical presentation and demographic data of all the patients. The study population was defined with the following inclusion criteria: emergency admission and complete occlusion of the abdominal aorta at the CTA exam. Primary aortic occlusion was defined, according to the literature, as aortic occlusion in the absence of atherosclerosis or aneurysmal disease or the presence of any aortic or vascular devices. Patients affected by the secondary form of AAO, as secondary to trauma, dissection, endovascular prothesis or graft infections, were excluded.

### 2.2. CT Imaging

CT examinations were performed using a multi-detector CT (MDCT) system (Aquilion 64, Toshiba Medical Systems, Fukuoka, Japan). All CT examinations were performed by volumetric spiral acquisition with a slice thickness of 1 mm with 0.625 reconstruction, a 512 × 512 matrix and a 40 × 40 cm FOV.

MDCT protocol includes an unenhanced CT scan of the thorax and abdomen extended to the proximal femoral vessels followed by the same acquisition package after contrast agent iv administration in the arterial (Angiography, CTA) and venous phase. MPR and Maximum Intensity Projection (MIP) were routinely used as an additional diagnostic tool.

Contrast agent consisted of 100–130 mL iodinated agent at high iodine concentration (370–400 mg/mL) injected at 4–5 mL/s, followed by 40 mL of saline at the same flow rate to obtain optimal vessel depiction.

We used automated bolus tracking to time the arterial phase, with region of interest (ROI) placed in the descending aorta at an attenuation threshold of 100 HU. In addition, we performed a portal phase (70 s delay from the end of the injection) and a delayed excretory phase (180 s delay) was detected for further evaluation in case of additional clinical suspects.

Informed consent was obtained from all patients at the time of the exam.

## 3. Results

From January 2019 to November 2022, 11 patients were diagnosed and treated at our hospital for abdominal PAO. Demographic data of the 11 patients (49 to 79 years old, mean age 65.27 years), are reported in Table 1. A total of 72.8% (8/11) of patients were males with a mean age of 64.5 years (age range from 49 to 79 years) and 27.2% (3/11) of patients were female with a mean age of 67.3 years (age range from 65 to 69 years). The male/female ratio was 2.66:1 while the global mean age was 65.27 years.

Laboratory tests demonstrated an increase in International Normalized Ratio (INR) in 4/11 (36.4%) patients, in Partial Thromboplastin Time (PTT) in 3/11 patients (27.3%) and in Fibrinogen in 5/11 (45.5%) patients. Fibrinogen was reduced in 3/11 (27.3%) patients. In addition, it was found that 10/11 (90.9%) patients had increased neutrophil leukocyte count and C-reactive protein (CPR) inflammatory factor. Screening of the thrombophilic pattern was negative for procoagulant alterations.

None of these patients showed any usual risk factors for atherosclerosis (hypertension, diabetes, dyslipidemia, coronary heart disease or cigarette smoking).

Nine out of eleven patients (81.8%) admitted to the Emergency Department were affected by acute pain, hypothermia and functional impotence of the lower limbs, one of the eleven (9.1%) patients was affected by acute lower limb paraplegia and one (9.1%) with acute abdominal pain (Table 2).

All 11 patients showed absence of lower limb pulses; they first underwent Doppler ultrasonography (US).

After the US confirmed the absence of pulses, they underwent CT angiography in ER settings of the thoraco-abdominal aorta extended to the proximal femoral vessels. No significant atherosclerotic change or any aneurismal dilation in the entire aorta was detected.

**Figure 1:**
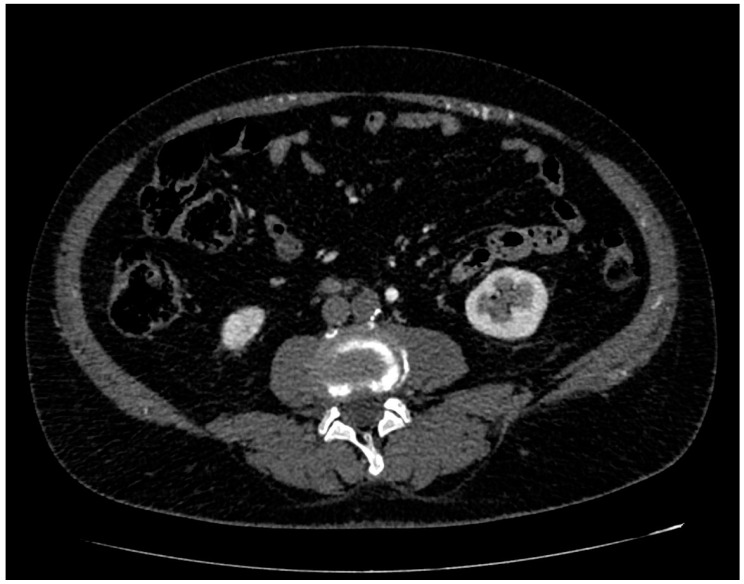
CT angiography, axial planes, arterial phase. The mesenteric inferior artery and its branches are normally enhanced while the aorta is completely occluded, in absence of signs of aneurysms and dissection.

**Figure 2:**
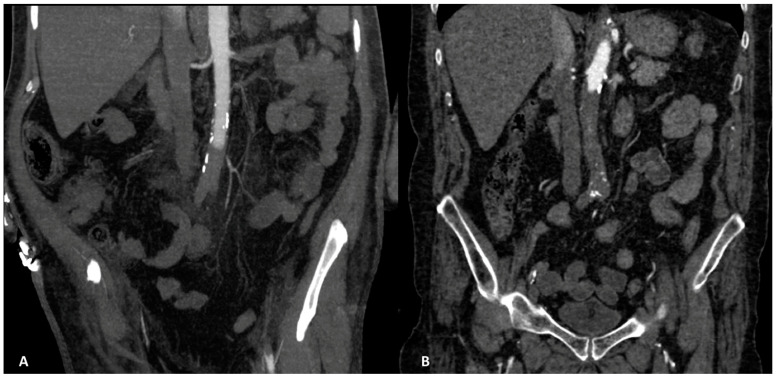
(**A**). CT angiography, coronal planes, arterial phase, MIP reconstruction. Aortic complete occlusion in the subrenal tract. Thrombosis located also in the left renal artery and extended until left common femoral artery can be noted (in our study found in 3/11 patients). (**B**). CT angiography, coronal planes, arterial phase. Aortic complete occlusion in the infrarenal tract, located at the origin of the right renal artery.

Aortic complete occlusion was detected in the subrenal tract in 9/11 cases (81.8%) and in the infrarenal tract in 2/11 patients (18.2%) (Figure 1 and Figure 2). In all patients, the occlusion extended through to the common iliac arteries bilaterally, and in 3/11 cases (27.3%) it also extended to the origin of the common femoral arteries (Table 3). Our results confirmed what had been previously published; that occlusion of the subrenal abdominal aorta through the aortoiliac bifurcation is the more common presentation [2,3,17,18,24].

**Figure 3:**
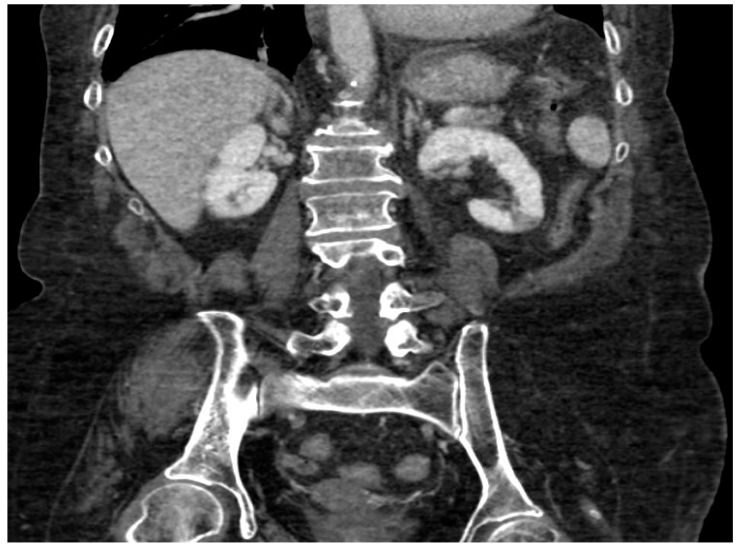
CT angiography, coronal planes, portal phase. Focal ischemia of the left renal parenchyma may be noted in the lower polar location.

**Figure 4:**
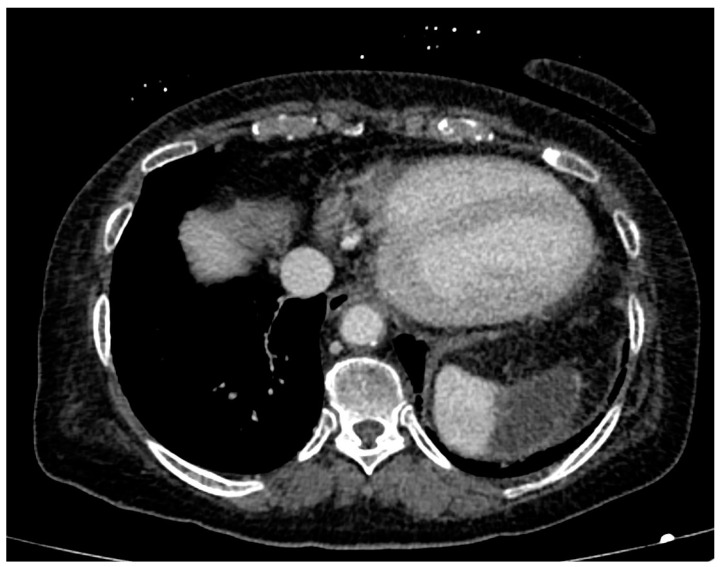
CT angiography, axial planes, portal phase. Focal ischemia of the splenic parenchyma may be noted in the upper anterior polar location.

**Figure 5:**
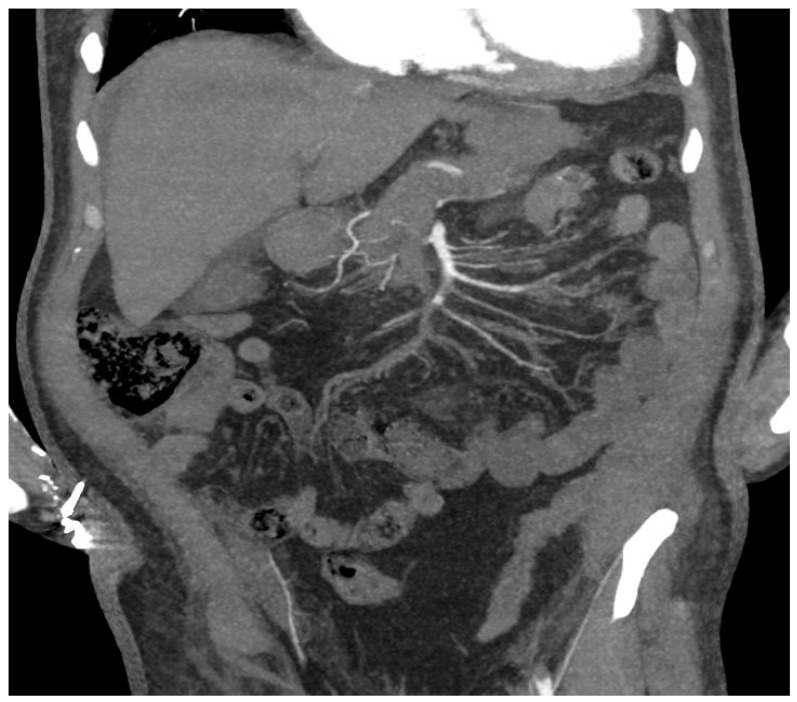
CT angiography, coronal planes, arterial phase, MIP reconstruction. Extended thrombosis in the superior mesenteric artery and its branches can be noted, with hypoperfusion of the intestinal loops.

The acute ischemic complications of abdominal parenchyma were:

- Focal ischemia of the left renal parenchyma in four patients (36.4%) (Figure 3);
- Focal ischemia of the splenic parenchyma in two patients (18.2%) (Figure 4);
- In one case (9.1%), hypoperfusion of the intestinal loops was evidenced due to extensive thrombosis of the superior mesenteric artery (Figure 5); this patient was the one with predominant abdominal pain symptoms.

In all these cases of parenchymal ischemia, no specific surgical treatment was performed.

After the CTA diagnosis, two patients (18.2%) were immediately transferred to the Intensive Care Unit (ICU) for acute severe worsening of clinical conditions. These patients died the same day of the CTA diagnosis in the ICU Department due to multi-organ failure (MOF).

In the other 9/11 patients (81.8%), successful surgical treatment was performed: 6/11 (54.5%) patients underwent successful aortoiliac embolectomy, 2/11 (18.2%) patients aortoiliac embolectomy + aorto-femoral bypass and 1/11 (9.1%) aortoiliac embolectomy + lower limb right amputation.

In addition, anticoagulation (warfarin, enoxaparin or dabigatran etexilate) and aspirin or clopidogrel was prescribed at discharge in all patients.

Two patients died during the early surgical follow-up (<30 days) for ischemic complications, due to MOF and high-grade acute renal failure, respectively. These two patients were admitted to the ICU of our hospital when they presented symptoms of the described complications.

The other patients who underwent surgery did not show any complications or recurrence during the entire follow-up (considered 1 year for all patients).

The early mortality rate was defined as post-surgical death from any cause within the first 30 days of follow-up, the overall mortality rate was defined as death from any cause in the patients included in the study and the effectiveness of surgical and medical therapy was defined as long-term survival (>1 year of follow-up).

In our study, the early mortality rate was 2/9 (22.2%), the overall mortality rate was 36.4% (4/11 patients) and the long-term survival, which was considered 1 year of follow-up, has resulted in 63.6% (7/11 patients).

## 4. Discussion

AAO is a rare and life-threatening condition that may cause arterial hypoperfusion or ischemia of the tributary parenchymatous organs or critical limb ischemia, depending on the extent and anatomical location of the thrombus. The etiology of this disease is multifactorial, depending on whether etiology is primary or secondary to other causes.

PAO is an extremely rare disease with an unclear incidence due to its infrequent occurrence [5,12,13,14,25].

As a definition, AAO is considered primary in etiology (PAO) when it develops in a regular aorta with no risk factors such as atherosclerotic occlusive or aneurysmal disease or devices. The etiology is still unknown; the interaction between host and environment factors may lead to the acute thrombosis of a non-pathologic abdominal aorta, and hypercoagulability disorders, immunologic and metabolic factors and toxicological cofactors may play an important role [1,16].

According to Verma et al., in the definition of PAO [26], none of our 11 patients had shown meaningful atherosclerotic change or any aneurismal dilation in the entire aorta nor any usual risk factors for atherosclerosis (cigarette smoking, hypertension, diabetes, dyslipidemia and/or coronary heart disease).

Although any significant difference between male and female genders has never been reported in the literature [3], in our study, we found that there was a mild prevalence in males (male/female ratio: 2.66/1). The mean age of our population was 65.27 years old, slightly older than what has previously been reported (<50 years old patients mean age) [27,28,29].

Verma et al. classified PAO in four types, from I to IV [26]:

- Type I: mural thrombus in the ascending and arch of the aorta (up to the origin of the left subclavian artery; Type Ia: limited to the ascending aorta, Type Ib: ascending aortic thrombus extending into the arch or aortic arch thrombus);
- Type II: mural thrombus in the descending thoracic aorta (distal to the left subclavian artery up to the coeliac artery); Type IIa: descending thoracic aorta thrombus above T8, Type IIb: descending thoracic aorta thrombus + supracoeliac aorta thrombus (T8-L1);
- Type III: mural thrombus in the aortic segment between the coeliac artery and the lower renal artery;
- Type IV: thrombus between the lower renal artery and the aortic bifurcation.

In addition, based on the thrombus morphology, each type of thrombus was further classified as sessile (S), pedunculated (P) or occlusive (O).

In our study, the distal abdominal aorta, intended from just below the origin of the renal arteries to the aorto-iliac bifurcation, was the tract most frequently involved in PAO [26]. According to Verma’s classification, 9/11 (81.8%) of our patients were classified as Type IV O and 2/11 (18.2%) patients as Type III O.

PAO clinical presentation is sudden and depends on the aortic occlusion level and thrombus localization. In our study, similarly to others [3,26,28,29], the majority of our patients (81.8%) were admitted to the Emergency Department with clinical signs and symptoms of acute onset of lower limb functional impotence, pain, hypothermia and ischemia. In these patients, the diagnosis can be suspected when the acute onset of lower limb symptoms is associated with the absence of femoral pulses, but in cases of paraplegia or sudden lower limb impotence, a correct differential diagnosis with neurological disease can be more complex and may delay the diagnosis. In the case of acute lower limb symptoms, the first clinical step should be the verification of peripheral femoral pulse palpation. If undetectable, MDCT angiography should be performed in order to distinguish neurologic from vascular causes that require immediate treatment [30].

Similar to other series [26,27,29], parenchymal ischemia was mostly asymptomatic but present at the time of the CTA examination and was treated conservatively because parenchymal ischemic areas were focal and not overly extensive.

**Figure 6:**
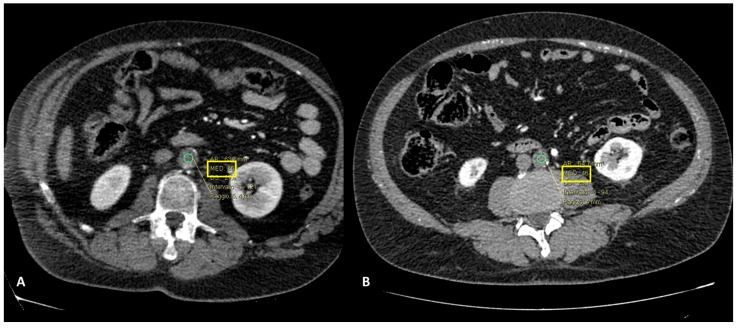
CT angiography, axial planes, arterial phase. In (**A**), the ROI placed in the aortic thrombus showed a value of 65 HU, while in (**B**), the value showed was 46 HU. Patient in (**A**) presented a chronic aortic occlusion while patient in (**B**) was one of the patients included in our study, affected by primary AAO (male, age 54 y/o).

Accurate diagnosis of PAO can be assessed by CTA, which is considered the first-choice imaging technique in emergency settings [21]. CTA allows for the direct anatomical visualization of the location of the occlusion, visualized as an endoluminal defect in the aortic lumen, and it allows the accurate assessment of concomitant occlusive disease affecting visceral arteries and the type and extent of collateralization, and supports an accurate surgical planning in the reaching of the affected vessel and the type of intervention to be carried out. CTA also provides insight into whether the thrombus is of recent onset, more hyperdense, or chronic, more hypodense (Figure 6A,B).

Combined with surgical treatment, anticoagulation is considered the first-line medical therapy [23] at the time of diagnosis, during surgical treatment and after at discharge, also combined with aspirin or clopidogrel.

The early mortality rate was 2/9 (22.2%), the overall mortality rate was 36.4% (4/11 patients) and the long-term survival, considered 1 year after follow-up, has resulted in 63.6% (7/11 patients). Despite the small case series in our study, we noted a low mortality rate after treatment, in line with similar studies, such as that of Crawford et al., which found an overall mortality of 34% and a 30-day mortality rate of 24% [4]. In conclusion, there are still several limits to our study; first of all, it was a retrospectively monocentric study; further, the reported sample was small, but we must also consider that the disease is extremely rare.

## 5. Conclusions

PAO is a rare entity with high morbidity and mortality rates if not recognized and treated promptly. Patients may present with acute onset of lower limb impotence, pain, hypothermia and, in severe cases, acute bilateral ischemia. Physicians should be aware to consider PAO also in absence of cardiovascular risks. A careful femoral pulse examination should be performed, and any pathological findings of femoral pulses should be investigated with CTA in order to differentiate cerebrovascular from neurologic from aortic causes. Aortic CTA is the first-choice imaging technique in emergency settings for the early diagnosis of this disease, providing insight into whether the occlusive thrombus is recent onset or a chronic, identifying concomitant occlusive disease affecting visceral arteries and its extent. CTA also supports accurate surgical planning: how to reach the affected vessel and the type of intervention to be carried out. AAO is infrequent. However, the diagnosis should not be difficult. The sudden onset of bilateral leg pain, neurologic deficits and lower extremity mottling should alert the physician to AAO.

Our study showed that a prompt diagnosis of PAO resulted in high overall survival and low mortality when immediate anticoagulation combined with surgical embolectomy and aorto-femoral bypass are used.

## Figures and Tables

**Table 1:** Demographic data of the patients included in the study in ascending order of age.

Patient	Sex	Age
1	M	49
2	M	54
3	M	55
4	M	63
5	F	65
6	F	68
7	M	68
8	F	69
9	M	74
10	M	74
11	M	79

**Table 2:** Clinical findings of the patients included in the study.

Patients n.	Prevalent Clinical Findings	%
9/11	Local pain, claudication, signs of limb ischemia	81.8%
1/11	Lower limb paraplegia	9.1%
1/11	Abdominal pain	9.1%

**Table 3:** Location of the aortic thrombotic occlusion.

Patients n.	Occlusive Thrombus Location	%
9/11	Subrenal aorta to common iliac arteries bilaterally	81.8%
2/11	Infrarenal aorta to common iliac arteries bilaterally	18.2%

## Data Availability

The data analyzed in the current study are available from the corresponding author upon reasonable request.
